# Joint Estimation of Mass and Center of Gravity Position for Distributed Drive Electric Vehicles Using Dual Robust Embedded Cubature Kalman Filter

**DOI:** 10.3390/s222410018

**Published:** 2022-12-19

**Authors:** Zhiguo Zhang, Guodong Yin, Zhixin Wu

**Affiliations:** 1 School of Mechanical Engineering, Southeast University, Nanjing 211189, China; 2 CATARC (Tianjin) Automotive Engineering Research Institute Co., Ltd., Tianjin 300300, China

**Keywords:** mass estimation, estimation of center-of-gravity, embedded cubature Kalman filter

## Abstract

The accurate estimation of the mass and center of gravity (CG) position is key to vehicle dynamics modeling. The perturbation of key parameters in vehicle dynamics models can result in a reduction of accurate vehicle control and may even cause serious traffic accidents. A dual robust embedded cubature Kalman filter (RECKF) algorithm, which takes into account unknown measurement noise, is proposed for the joint estimation of mass and CG position. First, the mass parameters are identified based on directly obtained longitudinal forces in the distributed drive electric vehicle tires using the whole vehicle longitudinal dynamics model and the RECKF. Then, the CG is estimated with the RECKF using the mass estimation results and the vertical vehicle model. Finally, different virtual tests show that, compared with the cubature Kalman algorithm, the RECKF reduces the root mean square error of mass and CG by at least 7.4%, and 2.9%, respectively.

## 1. Introduction

Traffic accidents cause a large number of casualties every year and precise vehicle motion control can effectively reduce the occurrence of traffic accidents [1]. Active safety systems are regarded as one of the most effective solutions for dealing with these traffic problems. The premise behind these systems is to obtain accurate model parameters [2]. These parameters can be collected through vehicle-to-vehicle communications [3] or state estimation methods [4]. Considering that the inertial and geometric parameters of distributed-drive electric vehicles can change significantly according to the actual load situation, it is necessary to implement online estimations of the vehicle model parameters. Accurate vehicle model parameters are critical not only for vehicle dynamics control but also for active vehicle safety technologies such as adaptive cruise control [5] and active obstacle avoidance systems [6]. Although the vehicle mass and center of gravity (CG) position are nominal values that can be directly obtained from the relevant vehicle manuals, they can change significantly when the vehicle load changes during actual driving. Considering that it is difficult for the existing onboard sensors to directly measure the mass and CG, more and more experts and scholars are using advanced filters and observers to indirectly estimate key parameters based on intelligent tires [7] or onboard sensors [8]. However, approaches based on intelligent tires are not widely applied due to their high cost and short life. Estimation methods based on onboard sensors are more popular.

Recursive least squares (RLS) with forgetting factors is a popular methodology employed for mass estimation. Zhang et al. [9] designed RLS with a double forgetting factor to identify mass and proved its effectiveness through tests. Kim et al. [10] designed a mass estimator based on longitudinal and lateral dynamics and using RLS. Chu et al. [11] used a filter to collect acceleration information to estimate the vehicle mass using the RLS algorithm. Wang et al. [12] used lateral dynamics and estimated the mass of a passenger vehicle using RLS. Lingman et al. [13] developed a mass estimation based on the traditional Kalman filter and achieved highly accurate estimations. Other similar methods were presented in [14,15,16]. Cai et al. [17] designed a two-layer mass estimation algorithm based on an extended Kalman filter and improved estimation accuracy. Lei et al. [18] identified vehicle mass using EKF. In addition, Torabi et al. [19] trained a neural network to predict vehicle mass. Korayem et al. [20] estimated the mass of a trailer using a feedforward neural network with 15 fully connected layers. However, these methods demand extremely high levels of completeness in the data set and are hardly applicable to a wide range of applications.

Similar to mass estimation, the estimation of the CG position is also a hot topic of research. Daniel et al. [21] proposed an RLS algorithm for predicting the CG position of a vehicle. Jounghee et al. [22] developed a vehicle vertical dynamics model for estimating the CG position. Muhammad et al. [23] used a five-degrees-of-freedom model to indirectly estimate the CG position using rotational inertia. Huang et al. [24] proposed a combined estimation method by fusing the adaptive Kalman filter with the EKF to achieve an accurate identification of the CG position of their vehicle.

The above approaches mainly estimate the mass and CG position separately and do not regard the influence of unknown measurement noise. In addition, state-of-the-art Kalman filtering algorithms were demonstrated to enhance the accuracy of vehicle state estimations, such as the cubature Kalman filter (CKF) algorithm [25]. Therefore, the estimation of mass and CG position with sophisticated Kalman filtering algorithms is an interesting direction for research. However, the conventional CKF algorithm still assumes that the measurement noise is known. To this end, a dual robust embedded cubature Kalman filter (RECKF) algorithm, which takes into account unknown measurement noise, is proposed for the joint estimation of mass and CG position.

This paper aims to propose a fusion estimation scheme to achieve the estimation of mass and CG position. Furthermore, we designed a RECKF estimator to reduce the effects of unknown noise on the performance of the estimation. Then we demonstrate the effectiveness of the proposed method through comparative experiments. The remainder of the paper is organized as follows: Section 2 presents the vehicle model; Section 3 describes the proposed joint estimation scheme in detail; Section 4 shows the test results and discussion; and finally, Section 5 summarizes the work.

## 2. Vehicle Model and Problem Formulation

**Figure 1:**
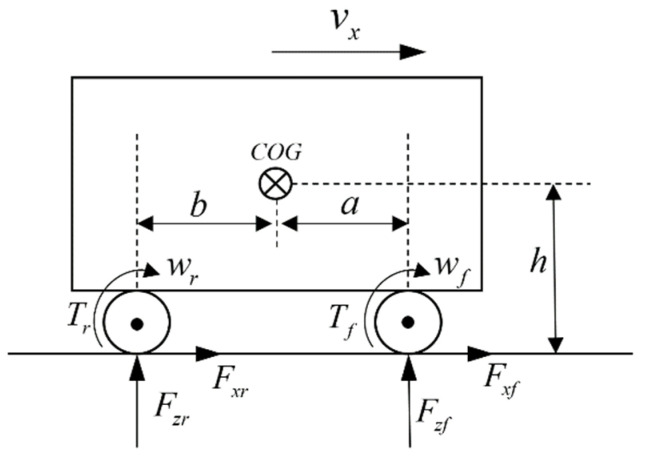
The vehicle model.

Considering that the estimation of mass and CG position does not involve the control of four-wheel drive force distribution, the vehicle model is simplified into a longitudinal motion model, and its dynamics model is shown in Figure 1.

With a known distributed drive electric vehicle wheel torque and angular speed, the longitudinal tire forces are given by

(1) $$F_{xf}=(T_{f}-J\omega_{f})/R\;$$

(2) $$F_{xr}=(T_{r}-J\omega_{r})/R\;$$

Without considering the effect of the ramp driving conditions during vehicle driving, the vehicle longitudinal dynamics equations are given by

(3) $$ma_{x}=(F_{xf}+F_{xr})-\frac{1}{2}C_{D}\rho Av_{x}^{2}-mgf_{roll}\;\;$$

(4) $$F_{zf}=\frac{mgb-mha_{x}}{\left(a+b\right)}$$

(5) $$F_{sf}=\frac{mga-mha_{x}}{\left(a+b\right)}$$

The meanings of specific vehicle model parameters are shown in Table 1.

The longitudinal force generated on each tire depends on the longitudinal slip and the normal force applied to the tire. In the low slip region, the longitudinal force generated by a single tire is proportional to its longitudinal slip or the linear part of the friction curve of the normal force. For all-wheel drive vehicles, the linear relationship between front and rear wheel slip and longitudinal forces can be expressed as

(6) $$F_{xi}=C_{xi}S_{i}{\hat{F}}_{zi}\;$$

(7) $$s_{i}=\frac{\omega_{i}R-v_{x}}{\max(\omega_{i}R,v_{x})}$$

where i=f,r denotes the vehicle’s front wheels and rear wheels respectively; $C_{xi}$ denotes the slope of the vehicle tire slip rate curve; $s_{i}$ denotes the vehicle tire slip rate. More details on the formulation of the linear tire model can be followed in [26].

In order to perform iterative estimations using discrete measurement signals and the RECKF, we need to transform Equations (1)–(7) into the form of a discrete state space.

(8) $$\left\{ \begin{array}{l} x_{k+1}=f(x_{k},u_{k})+w_{k}\\ z_{k+1}=h(x_{k+1},u_{k+1})+v_{k+1} \end{array}\right.$$

where the parameters are presented in Table 2.

For mass estimation, x=[m],$z=[a_{x}]$; For estimation of CG position, x=[a],$z=[F_{z}]$. $F_{z}$ is the vertical force of the tire.

## 3. Methodology

**Figure 2:**
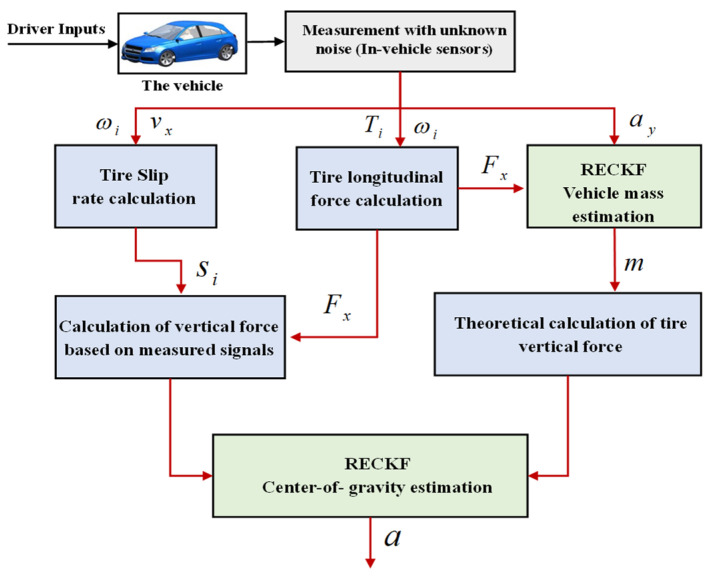
The framework of the estimation method.

As shown in Figure 2, the mass is first estimated using information such as lateral acceleration and longitudinal tire force using the RECKF algorithm. Then the vehicle tire slip rate is computed from the longitudinal vehicle speed and wheel speed according to Equation (7). Next, the measured value of tire vertical force is obtained according to Equation (6), while the theoretical value of tire vertical force is computed according to Equations (4) and (5). Finally, the measured value and the theoretical values of tire vertical force are used as input for the RECKF to estimate the CG position.

### 3.1. The RECKF

The conventional CKF method enhances the accuracy of the state estimation but does not account for the impact of unknown statistical properties of the noise. To further improve the nonlinear fit of the CKF, an embedded CKF is used first for the vehicle state estimation [3] and this achieves a favorable estimation performance. Inspired by this work, we propose a RECKF to estimate the vehicle model parameters. The iterative steps of the RECKF are given by

(1) Initialization:

(9) $${\hat{x}}_{0}=E(x_{0})$$

(10) $$P_{0}=E[(x_{0}-{\hat{x}}_{0}){\left(x_{0}-{\hat{x}}_{0}\right)}^{T}]$$

where *E* means to perform the mathematical expectation calculation, and P denotes the error covariance matrix of x.

The embedded cubature sampling points $\vartheta_{i}$ and weight *ϕ_i_* are given by.

(11) $$\vartheta_{i}={\left[0\right]}_{i}\;\;\;\;\;i=1;\;\;\;\;\;\;\;\;\;\vartheta_{i}=e_{i}=[eye(n),-eye(n)]\sqrt{2}\rho \;\;,\;\;\;\;\;i=2,3,\cdots 2^{n}+1$$

(12) $$\phi_{i}=1-\frac{1}{2\rho^{2}}\;\;\;\;\;\;\;\;\;\;i=1;\phi_{i}=\frac{1}{2^{n+1}\rho^{2}}\;\;\;\;\;\;\;\;\;\;i=2,3,\cdots 2^{n}+1$$

where *n* is the dimension of x, and ρ is a constant. Some specific details of the formulation are given in [27].

(2) Time prediction:

Singular value decomposition of $P_{k-1/k-1}$.

(13) $$P_{k-1/k-1}=U\left[\begin{array}{l} S\;\;\;\;\;\;0\\ 0\;\;\;\;\;\;\;0 \end{array}\right]V^{T}$$

(14) $$P_{k-1/k-1}=U_{k-1/k-1}S_{k-1/k-1}V_{k-1/k-1}^{T}$$

where *S* represents a diagonal matrix, and $P_{k-1/k-1}$ is the symmetric covariance matrix.

Evaluate the embedded cubature points

(15) $$\chi_{k-1/k-1}^{\left(i\right)}=S_{k-1/k-1}^{}\xi_{i}+{\hat{x}}_{k-1/k-1}^{}$$

where ${\hat{x}}_{k-1/k-1}$ is a priori estimated value. $\chi_{k-1/k-1}^{\left(i\right)}$ is an embedded cubature point of ${\hat{x}}_{k-1/k-1}$.

Evaluate the propagated embedded cubature points

(16) $$\chi_{k/k-1}^{*(i)}=f(\chi_{k-1/k-1}^{\left(i\right)},u_{k-1})$$

Evaluate ${\hat{x}}_{k/k-1}$ and $P_{k/k-1}$

(17) $${\hat{x}}_{k/k-1}^{}=\sum_{i=1}^{c}\omega_{i}\chi_{k-1/k-1}^{*(i)}$$

(18) $$P_{k|k-1}=\sum_{i=1}^{c}\omega_{i}\chi_{k/k-1}^{*(i)}\chi_{k/k-1}^{*(i)T}-{\hat{x}}_{k/k-1}^{}{\hat{x}}_{k/k-1}^{T}+Q_{k-1}$$

(3) Measurement prediction:

Singular value decomposition of $P_{k/k-1}$

(19) $$P_{k/k-1}=U_{k/k-1}S_{k/k-1}V_{k/k-1}^{T}$$

Evaluate the embedded cubature points

(20) $$\chi_{k/k-1}^{\left(i\right)}=S_{k/k-1}^{}\xi_{i}+{\hat{x}}_{k/k-1}^{}$$

Calculate the propagated embedded cubature points of the measurement vector

(21) $$Z_{k/k-1}^{\left(i\right)}=h(\chi_{k/k-1}^{\left(i\right)},u_{k})$$

Evaluate ${\hat{z}}_{k/k-1}^{}$, the innovation covariance matrices $P_{zz,k/k-1}$, and the cross-covariance matrix $P_{xz,k/k-1}$

(22) $${\hat{z}}_{k/k-1}^{}=\sum_{i=1}^{c}\omega_{i}Z_{k-1/k-1}^{\left(i\right)}$$

(23) $$P_{zz,k|k-1}=\sum_{i=1}^{c}\omega_{i}Z_{k/k-1}^{\left(i\right)}Z_{k/k-1}^{(i)T}-{\hat{z}}_{k/k-1}^{}{\hat{z}}_{k/k-1}^{T}+R_{k}$$

(24) $$P_{\mathrm{xz},k|k-1}=\sum_{i=1}^{c}\omega_{i}\chi_{k/k-1}^{\left(i\right)}Z_{k/k-1}^{(i)T}-{\hat{x}}_{k/k-1}^{}{\hat{z}}_{k/k-1}^{T}$$

The gain matrix $W_{k}$ and the posterior state ${\hat{x}}_{k/k}$ are given by

(25) $$W_{k}=P_{\mathrm{xz},k|k-1}P_{zz,k|k-1}^{-1}$$

(26) $${\hat{x}}_{k/k}^{}={\hat{x}}_{k/k-1}^{}+W_{k}(z_{k}-{\hat{z}}_{k|k-1})$$

According to the relevant conclusions in the literature [28], the error covariance matrix considering the unknown measurement noise is as follows

(27) $$P_{k/k}={\left(P_{k/k-1}^{-1}+P_{k/k-1}^{-1}P_{xz,k/k-1}R_{k}^{-1}P_{xz,k/k-1}^{T}P_{k/k-1}^{-T}\;-r^{-2}I_{n}\right)}^{-1}$$

where r is a constant to be determined according to the specific object of study.

### 3.2. The Flowchart of Joint Estimation

**Figure 3:**
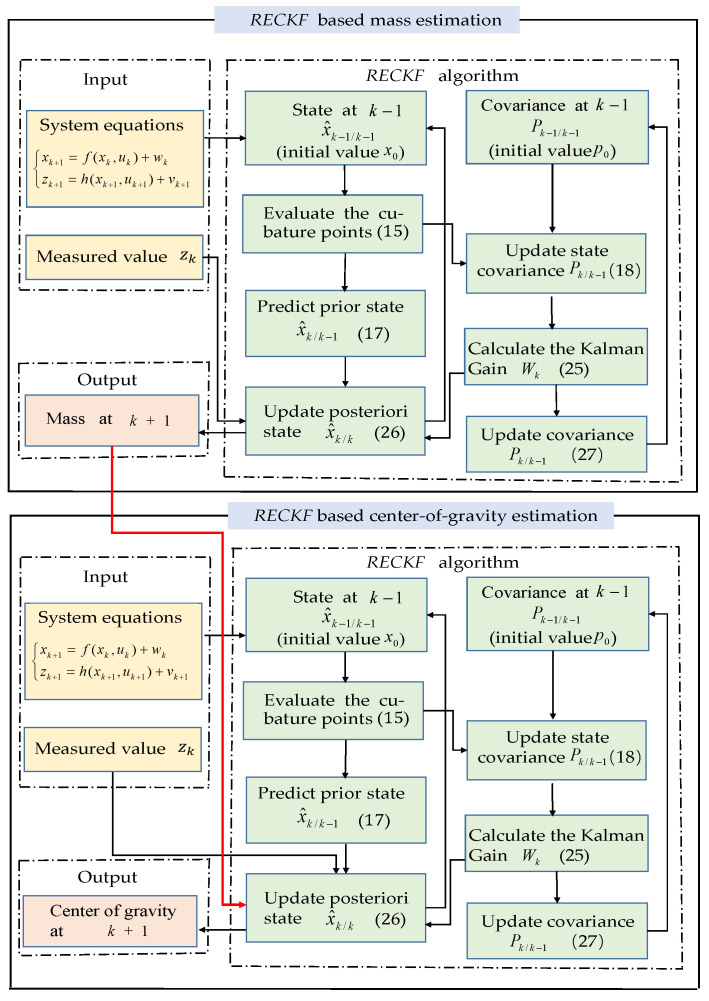
The flowchart of joint estimation.

Figure 3 depicts the RECKF-based vehicle mass and CG position estimation flowchart. The RECKF is first used for mass estimation, and then the output of this RECKF is used as the input for another RECKF to estimate the CG position in real-time. The specific iterative process of the RECKF is shown in the green box.

## 4. Results and Discussion

To verify the effectiveness of the RECKF, a co-simulation platform of Carsim and Matlab/Simulink was established to conduct simulation experiments under two different conditions of acceleration and deceleration. The superiority of the RECKF is further verified by comparing it with the traditional CKF algorithm. The vehicle model parameters are listed in Table 3.

### 4.1. Acceleration Test

**Figure 4:**
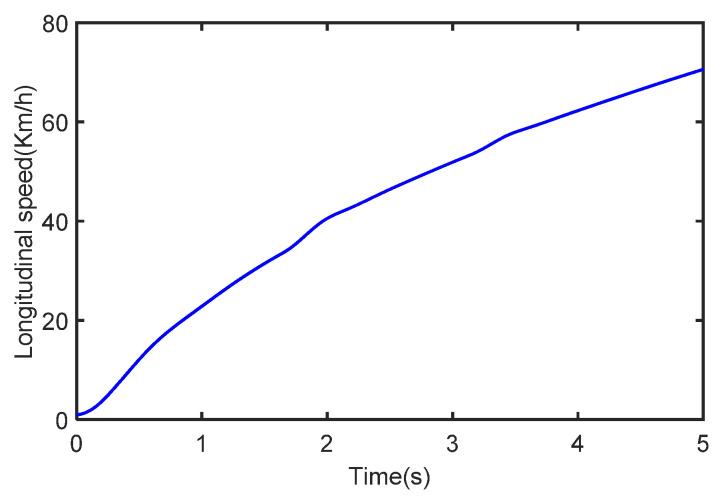
The vehicle speed in the case of acceleration.

**Figure 5:**
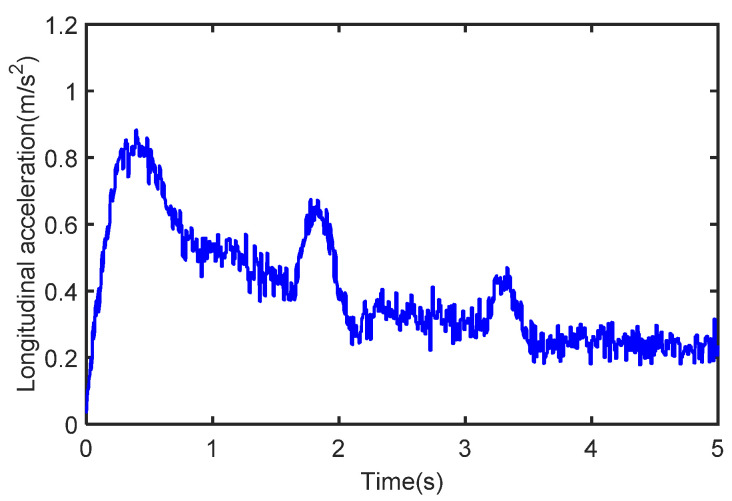
The acceleration in the case of acceleration.

The initial vehicle velocity is set to 1 km/h, the throttle opening is 40%, the process noise covariance is known, and the measurement noise is unknown. During the whole estimation process, the vehicle speed and longitudinal acceleration are shown in Figure 4 and Figure 5.

**Figure 6:**
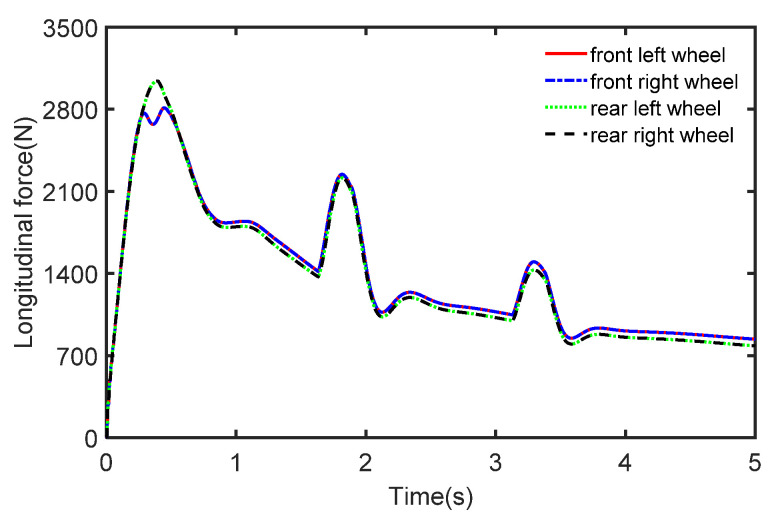
The tire forces in the case of acceleration.

It can be seen that the vehicle speed varies with the magnitude of acceleration, and the real measurement of the sensor is simulated by adding Gaussian white noise to the acceleration. The longitudinal force curve of the vehicle is shown in Figure 6 which shows that the distributed drive electric vehicle has driving force at all four wheels under acceleration conditions.

**Figure 7:**
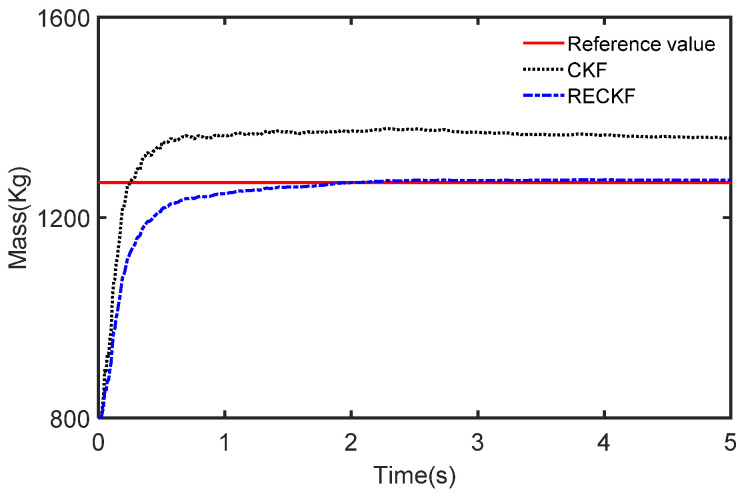
The estimated mass in the case of acceleration.

**Figure 8:**
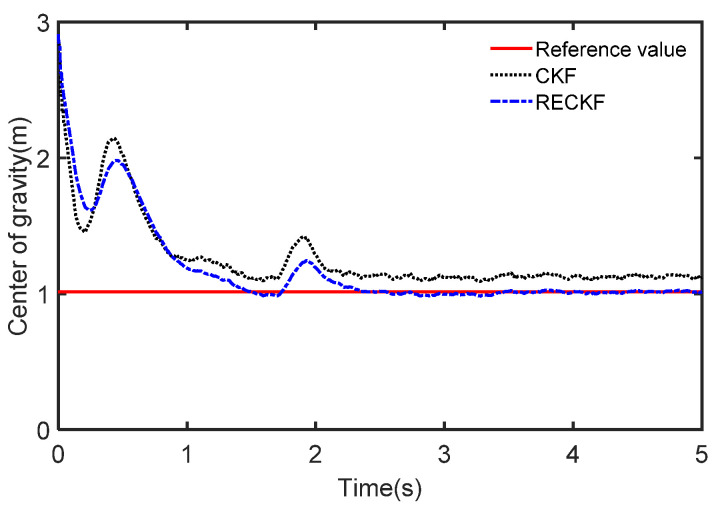
The estimated CG in the case of acceleration.

The results of the different methods used to estimate the mass are presented in Figure 7 where the reference value of mass is 1270 Kg. We can see that the CKF-based estimation curve rises faster at the beginning but the final stable value is much larger than the reference value. In contrast, the RECKF-based estimation curve tracks the reference value well. This is because the RECKF can suppress the effect noise has on the mass estimation accuracy. To further demonstrate the joint estimation effect, the CG position estimation curve is shown in Figure 8. The reference value is set to 1.015. The mass estimates obtained from the CKF and RECKF in Figure 7 are used as inputs to estimate the CG position. It can be seen from Figure 8 that the RECKF is closer to the reference value. The root mean square error metric (RMSE) is applied to further compare the estimation accuracy of the two algorithms, as shown in Table 4. It can also be seen that the estimation performance of RECKF is better than that of CKF. Specifically, the RMSE for mass estimation is reduced by 30.9% and the REMS of the CG position estimation is reduced by 2.9%.

### 4.2. Deceleration Test

**Figure 9:**
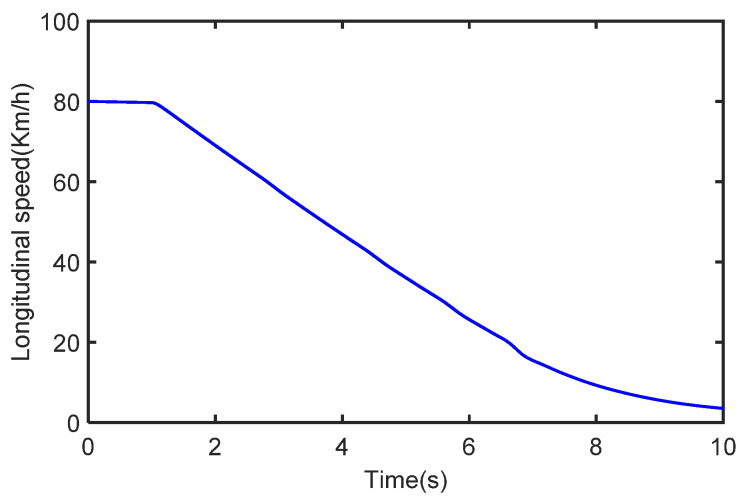
The vehicle speed in the case of deceleration.

**Figure 10:**
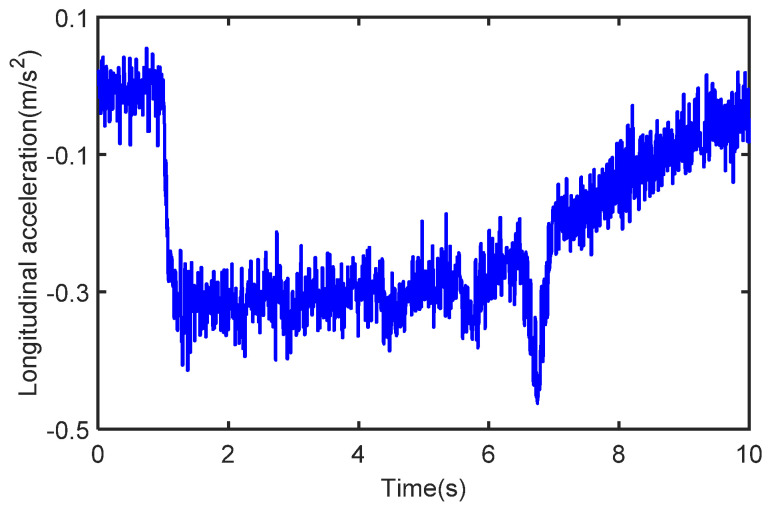
The acceleration in the case of deceleration.

**Figure 11:**
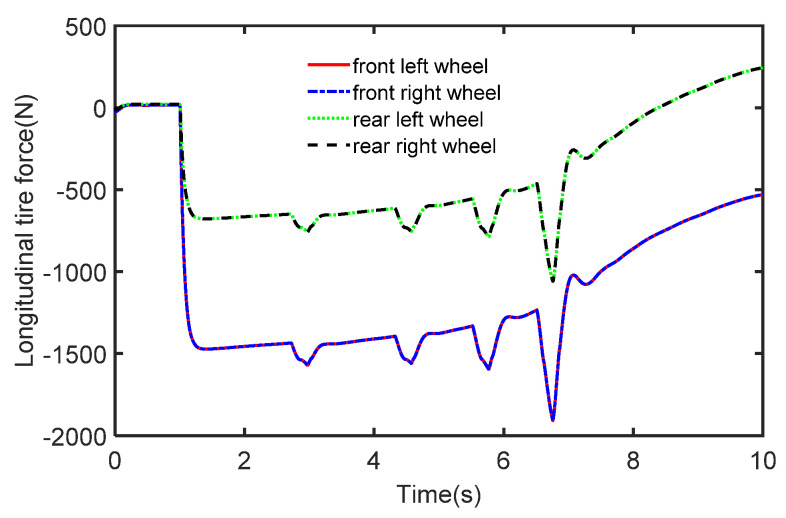
The tire forces in the case of deceleration.

The initial vehicle speed is 80 km/h and the braking operation is applied to the vehicle after 1 s. The process noise covariance statistics are known, and the measurement noise statistics are unknown. The estimated process vehicle speed and longitudinal acceleration are shown in Figure 9 and Figure 10. It can be seen that the vehicle acceleration is positive for the first 1 s, after which, the vehicle’s speed starts to drop with the braking intervention, and the vehicle acceleration falls into the negative. It can be seen that the vehicle speed varies with the magnitude of the acceleration, while the real sensor measurements are simulated by adding Gaussian white noise to the acceleration. In Figure 11, we can see that the longitudinal force curve of the vehicle is not equal for the front and rear braking forces. This indicates that the braking force distribution system is working in real-time to ensure the stability of braking.

**Figure 12:**
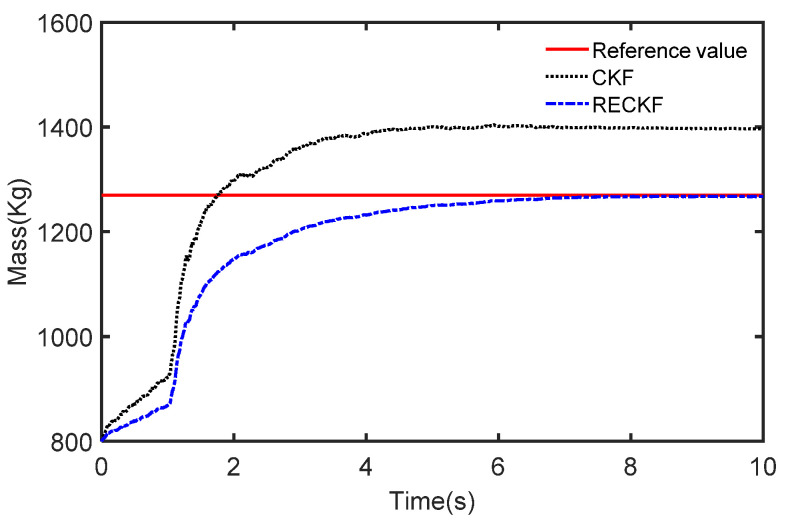
The estimated mass in the case of deceleration.

The results of the different methods used to estimate the mass are presented in Figure 12. The curves slowly rise from 800 Kg in the first 1 s, and with the addition of braking, the curves start to rapidly rise to track the reference value. However, it can also be seen that for the CKF at the beginning the rise is fast but the final stable value is much larger than the reference value. This is due to the unknown measurement noise resulting in a decrease in the estimation accuracy of the CKF. Furthermore, the RECKF is able to track the reference value and it fits well.

**Figure 13:**
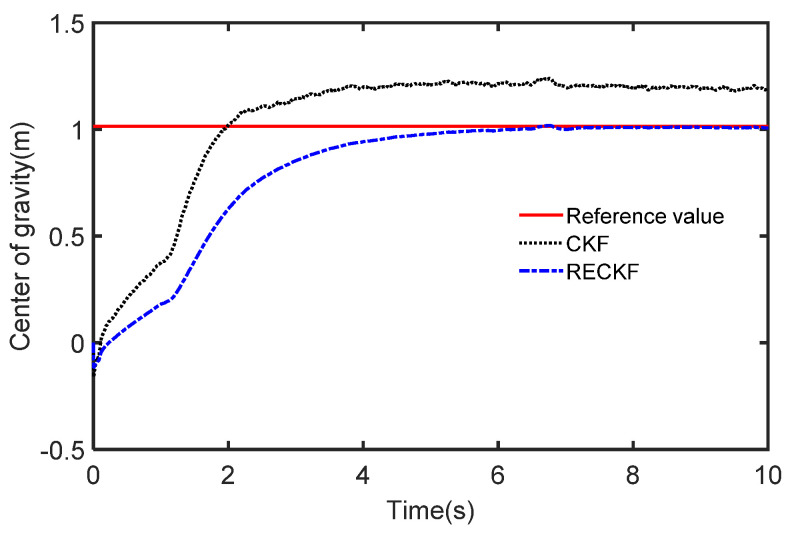
The estimated CG in the case of deceleration.

To further demonstrate the joint estimation effect, the CG position estimation curve under acceleration conditions is shown in Figure 13. It can be seen from Figure 13 that the RECKF is closer to the reference value. The RMSE is used to further compare the accuracy of the two algorithms, as shown in Table 5. Also, the estimation error of the RECKF is smaller than that of CKF. Specifically, the RMSE for mass estimation is reduced by 7.4% and the REMS of the CG position estimation is reduced by 12.6%.

## 5. Conclusions

In this paper, a novel joint estimation scheme is proposed to achieve the estimation of mass and CG position. This framework contains two RECKF estimators to identify mass and CG respectively, where the RECKF is a new estimator that combines robust filtering and an ECKF to suppress the influence of unknown noise. The experimental results of the virtual tests show that the proposed estimation scheme can achieve a simultaneous estimation of multiple parameters with high estimation accuracy. On the other hand, the RECKF can suppress the influence of unknown noise on the estimation accuracy. The proposed method can be used not only for passenger vehicles but also for commercial vehicles or intelligent vehicles. In our study, the effect of road slope was not considered, and the fusion estimation of mass, CG position, and slope will be carried out in the next step to further improve the identification accuracy of the parameters. Due to limited resources in some of the objective conditions, we have not conducted real vehicle experiments. We will conduct real vehicle experiments in the future when equipment and space are available.

## Figures and Tables

**Table 1:** The parameters of the longitudinal motion model.

Symbol	Description
m	vehicle mass
a	distance from the center of gravity to the front axle
b	distance from the center of gravity to the rear axle
$v_{x}$	longitudinal velocity
$T_{r}$	driving moments of the rear wheels
$T_{f}$	driving moments of the front wheels
$F_{xf}$	longitudinal forces at the front wheels
$F_{xr}$	longitudinal forces at the rear wheels
$F_{zf}$	vertical forces at the front wheels
$F_{sr}$	vertical forces at the rear wheels
h	height of the CG
J	wheel inertia
$\omega_{r}$	rear wheel speeds
$\omega_{f}$	front wheel speeds
R	tire radius
$C_{D}$	air drag influence coefficient
ρ	air density
A	windward area
g	weight acceleration
$f_{roll}$	rolling resistance coefficient
$a_{x}$	longitudinal acceleration

**Table 2:** The parameters of Equation (8).

Symbol	Variables
z	measurement vector
f	state transition function
u	input vector
w	process noise
h	output function
v	measurement noise

**Table 3:** The parameters of the vehicle model.

Symbol	Values
m	1270 kg
a	1.015 m
A	2.2 m
$I_{z}$	$1536.7\;kg\cdot m^{2}$
b	1.895 m
$C_{xf}$	28

**Table 4:** The RMSE in the case of acceleration.

Symbol	CKF	RECKF
*m*	111.20	76.76
*a*	0.3691	0.3586

**Table 5:** The RMSE in the case of deceleration.

Symbol	CKF	RECKF
*m*	242.33	224.44
*a*	0.5330	0.4663
